# Capturing the Dynamics of the Social Environment Through Experience Sampling Methods, Passive Sensing, and Egocentric Networks: Scoping Review

**DOI:** 10.2196/42646

**Published:** 2023-03-17

**Authors:** Anna M Langener, Gert Stulp, Martien J Kas, Laura F Bringmann

**Affiliations:** 1 Groningen Institute for Evolutionary Life Sciences Groningen Netherlands; 2 Department of Sociology, Faculty of Behavioural and Social Sciences, University of Groningen & Inter-University Center for Social Science Theory and Methodology Groningen Netherlands; 3 Department of Psychometrics and Statistics, Faculty of Behavioural and Social Sciences, University of Groningen Groningen Netherlands; 4 Interdisciplinary Center Psychopathology and Emotion Regulation, University Medical Center Groningen, University of Groningen Groningen Netherlands

**Keywords:** social context, experience sampling method, egocentric network, digital phenotyping, passive measures, ambulatory assessment, mobile phone

## Abstract

**Background:**

Social interactions are important for well-being, and therefore, researchers are increasingly attempting to capture people’s social environment. Many different disciplines have developed tools to measure the social environment, which can be highly variable over time. The experience sampling method (ESM) is often used in psychology to study the dynamics within a person and the social environment. In addition, passive sensing is often used to capture social behavior via sensors from smartphones or other wearable devices. Furthermore, sociologists use egocentric networks to track how social relationships are changing. Each of these methods is likely to tap into different but important parts of people’s social environment. Thus far, the development and implementation of these methods have occurred mostly separately from each other.

**Objective:**

Our aim was to synthesize the literature on how these methods are currently used to capture the changing social environment in relation to well-being and assess how to best combine these methods to study well-being.

**Methods:**

We conducted a scoping review according to the PRISMA (Preferred Reporting Items for Systematic Reviews and Meta-Analyses) guidelines.

**Results:**

We included 275 studies. In total, 3 important points follow from our review. First, each method captures a different but important part of the social environment at a different temporal resolution. Second, measures are rarely validated (>70% of ESM studies and 50% of passive sensing studies were not validated), which undermines the robustness of the conclusions drawn. Third, a combination of methods is currently lacking (only 15/275, 5.5% of the studies combined ESM and passive sensing, and no studies combined all 3 methods) but is essential in understanding well-being.

**Conclusions:**

We highlight that the practice of using poorly validated measures hampers progress in understanding the relationship between the changing social environment and well-being. We conclude that different methods should be combined more often to reduce the participants’ burden and form a holistic perspective on the social environment.

## Introduction

### Background

Humans are fundamentally social beings. The lack of social interactions and social relations is often associated with poor mental well-being [1-3]. Although positive social relationships can enhance mental well-being, negative relationships are found to be a risk factor for various psychological and neuropsychiatric disorders [4-7]. In addition, various psychological disorders are characterized by difficulties in engaging in social interactions [8]. Accordingly, clinical therapists and researchers are increasingly attempting to capture the social interactions of their patients to improve treatment outcomes [9,10].

A general question that psychology and other disciplines face in this quest is how to best capture people’s social environment and its effect on an individual’s well-being. This question has become more pertinent in recent years, in which research has recognized that important psychological processes are dynamic, implying that emotions and (social) behavior fluctuate over time, and static measures do not suffice in capturing these dynamics [11]. This insight has led to the development of several technologies in different disciplines that are able to capture the changing social environment, for example, day-to-day social interactions. In total, 3 methods are, to the best of our knowledge, currently most often used to assess the changing social environment occurring in daily life, namely, the experience sampling method (ESM) [12], passive sensing (including the electronically activated recorder [EAR]) [13,14], and egocentric networks [15]. The development and implementation of these methods have thus far occurred largely separately from each other.

In psychology, ESM is used as a standard tool to capture how psychological processes evolve over time. It offers great potential as it provides a picture of daily emotions and (social) behavior [16]. Participants receive a device to fill out brief daily questionnaires when a signal occurs. For example, participants fill out a questionnaire on an app after a push notification is sent.

Given that ESM can be burdensome for the participants, different disciplines have started to use passive sensing, which collects data via sensors from smartphones or other wearable devices (eg, smartwatches). This allows for monitoring participants constantly over time. For instance, smartphones can track an individual’s whereabouts (through a GPS), social engagement (through the use of social media apps), and social interactions (by measuring people’s conversations using the microphone [17,18]). A subgroup of studies that use sensors to passively collect data are EAR studies. In EAR studies, brief snippets of environmental sounds are recorded to investigate the activities or emotions of a person [14,19]. Emotions can be assessed by transcribing the audio snippet and using Linguistic Inquiry and Word Count [20]. Linguistic Inquiry and Word Count is a dictionary that calculates the percentages of included words for different categories. Some categories describe the emotional tone someone used while communicating, such as positive and negative affect [21].

Sociologists often use repeated egocentric networks to investigate how social connections change over time, for example, whether the occurrence of mental illness leads to network attrition, which means that fewer social contacts are included in the social network [22], or how the social network of a person changes during substance abuse disorder recovery [23]. To collect egocentric networks, researchers typically ask so-called name-generating questions to identify the important social contacts of a person, which are also called alters. For example, a name-generating question could be “Please list 25 names of individuals...with whom you have had contact in the last year” [24]. After alters are identified, further questions about the characteristics of those alters and the characteristics of the relationship to those alters are asked (eg, age of the alter, frequency of contact, and how close someone is to the alter) [15]. Overall, egocentric networks represent relationships (ties) between a specific individual (ego) and connected persons (alters). When relationships between alters are assessed, structural properties and composition effects of the ego’s network can be calculated, such as density (the proportion of existing ties relative to all possible ties) [15], which has been shown to affect ego’s health outcomes [25]. Similar to ESM, egocentric networks are burdensome to collect [26,27], for example, compared with passive measures.

These tools are likely to tap into different parts of people’s changing social environment. In this paper, we broadly focus on all aspects of the social environment that differ for each person and vary over short time scales (eg, minutes, days, and weeks). We include the behavioral and psychological levels, also known as the structural and cognitive levels [28,29]. The behavioral level indicates both what people do and the structural aspects of the social network (eg, network size), whereas the psychological level shows, for instance, what people feel or think [28,29]. For example, a person can have frequent social contact (behavioral level) and still feel lonely (psychological level) [4]. Therefore, we consider both levels (psychological and behavioral) as important to capture the full changing social environment.

### Objectives

So far, it remains unclear which parts of the changing social environment ESM, egocentric networks, and passive sensing attempt to capture in the literature and whether and where there is overlap. Therefore, the first question that we addressed is which aspects of the behavioral and psychological levels of the changing social environment are measured using the 3 different techniques. This question is important in light of the call for a better conceptualization of (psychological) constructs to advance theory and measurement [30-32]. We provided a way forward in how the changing social environment can be conceptualized by showing which period each method captures and which aspects of the social environment different methods tap into. This will also advance the understanding of mental health as it provides an overview of which aspects of the changing social environment are currently studied in relation to well-being.

For each of these methods, it is important to consider whether they measure the concept that they are intended to measure. Thus, the second question that we addressed is if and how the measures are validated. This second question mirrors a recent debate that psychological research is facing, namely, the problems associated with the use of nonvalidated questionnaires [33]. We investigated whether this also applies to measures that are used to capture the social environment.

Finally, ESM, egocentric networks, and passive sensing come from different disciplines and, therefore, are likely to be used separately from each other. However, we believe that a holistic understanding of the dynamic relationship between mental health and the changing social environment requires insights from different aspects that are measured using the different tools. We do not know how often these tools have been combined in previous research and how they can be combined optimally. Consequently, the last question that we addressed is how the measures have been combined in previous research.

## Methods

### Protocol

We conducted a scoping review [34]. Although this was not a systematic review, we designed and wrote this study according to the PRISMA (Preferred Reporting Items for Systematic Reviews and Meta-Analyses) statement. The PRISMA checklist can be found in Multimedia Appendix 1 [35]. This review was not registered before conducting it.

### Literature Search Strategy

We aimed to identify studies that used methods that were able to capture the changing social environment in relation to well-being in an adult population. Therefore, we conducted a systematic search to identify studies that used ESM, passive sensing, or repeated egocentric networks to measure the changing social environment in relation to well-being or psychological disorders. The final database search was completed in July 2021. We conducted searches in Web of Knowledge, PsycINFO, and PubMed with no date limitation. We created 2 different search strings and ran each search string in each database to identify studies of interest. Both full search strings can be found in Multimedia Appendix 2 [36-38].

### Eligibility Criteria

For inclusion, studies must have (1) used ESM, passive sensing, or repeated egocentric networks to measure the changing social environment (we included all studies that broadly focused on aspects of the social environment, which varied over short time scales [eg, minutes, days, and weeks], as well as studies that focused on behavioral aspects [such as social interactions] and psychological aspects [such as social support or relationship characteristics]); (2) measured the social environment in relation to well-being, psychological disorders, or psychiatric disorders; (3) measured the social environment in an adult population without nonpsychological medical conditions; (4) studied the social environment in the daily life of a participant; and (5) been published in English.

Consequently, we excluded studies that focused on children or older adults and medical treatment evaluations of nonpsychological or psychiatric disorders. Older adults were identified as such if the study explicitly said that it focused on, for example, “old age,” “older women,” or “older people” [39-41]. We also excluded studies that focused on medical conditions (eg, stroke). Furthermore, we excluded studies that only focused on the social environment in an occupational setting or on dyadic interactions as we were interested in methods that capture the broad social environment in daily life (see Multimedia Appendix 3 for a more extensive list).

The eligibility of the studies was assessed by 1 researcher (AML). A random subsample of studies (n=21) at the beginning of the coding process was independently assessed by 2 researchers (AML and LFB) to formulate eligibility criteria and discuss discrepancies with the research team.

### Data Extraction

We extracted information about the study (year of publication and objective), sample characteristics (sample size, sample population, and country of residence), procedure (study length and assessment frequency), statistical analysis, validation, and which sensors were used in passive sensing studies.

We further extracted information on which questions were used to capture the social environment. The questions used in the selected ESM and egocentric network studies were diverse and, therefore, hard to analyze. Thus, we developed categories that summarized these questions. We developed those categories as an iterative process. After reading 15 studies, we summarized the measures of the social environment that were repeatedly used across the studies. While reading more papers, we revised these categories. In total, we identified 12 categories for ESM and 5 categories for egocentric networks, which are reported in the *Results* section. We created the category “Other” for items that did not fit in any of the other categories.

Data extraction was conducted by 1 researcher (AML). The categories were developed together, and specific cases were discussed within the research team to increase the clarity of the categorization of the studies.

### Risk of Bias

Our primary aim was to identify studies that used measures to capture the changing social environment. Similarly to O’Donohue et al [42], we argue that the outcome and quality of the studies were less relevant and would not have affected the eligibility of the studies as no hypothesis was tested. Therefore, the risk of bias for each study was not assessed. However, the validity of the measures used was examined and is described in the *Question 2: How Well Are the Measures of the Social Environment Validated?* section.

## Results

### Overview of the Selected Studies

**Figure 1:**
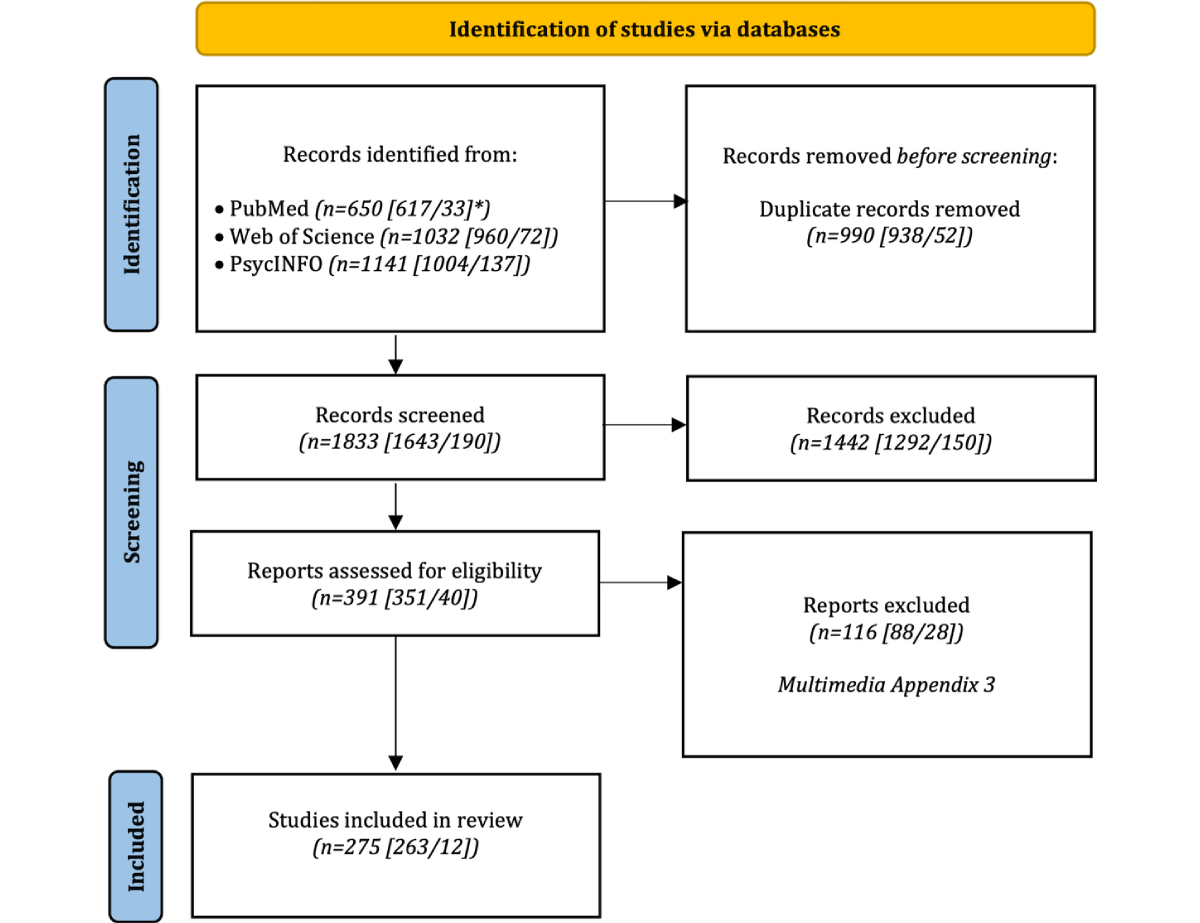
Flow diagram of the included studies (adapted from Page et al [35]). *The first number indicates the records that were identified using the first search string, which aimed to extract studies that used the experience sampling method or passive sensing, whereas the second number indicates the records that were identified using the second search string, which aimed to extract studies that used repeated egocentric networks.

We identified 1833 articles that measured the social environment using ESM, passive sensing, or repeated egocentric networks after removing duplicates (Figure 1). If we decided to exclude a study during the screening process, we indicated a reason for doing so. This overview can be found in Multimedia Appendix 3. We removed 78.67% (1442/1833) of the studies after title and abstract screening. In total, 29.7% (116/391) of the studies were excluded after reviewing the full text. Thus, we included 275 studies in our scoping review.

Most of the included studies only used 1 method, meaning that studies only used ESM (223/275, 81.1%), passive sensing (19/275, 6.9%), or egocentric networks (12/275, 4.4%). Note that some of the identified papers included multiple studies, which leads to a total of 238 studies that only used ESM, 20 studies that only used passive sensing, and 12 studies that only used egocentric networks.

In total, 5.5% (15/275) of the studies combined ESM and passive sensing to measure the social environment. Repeatedly assessed egocentric networks were not combined with other methods. However, 2.2% (6/275) of the studies combined egocentric networks that were assessed only once with ESM (4/6, 67%) or passive sensing (2/6, 33%).

### Study Characteristics

Multimedia Appendix 3 provides an overview of the individual characteristics of each study. The studies were published between 1987 and 2021. The studies took place mainly in the United States (178/275, 64.7%) and Europe (80/275, 29.1%).

On average, 201 (SD 706; median 102) participants took part in the 275 selected studies. More than half of the studies included (healthy) students (105/275, 38.2%) or adults (63/275, 22.9%) as participants. In total, 2.2% (6/275) of the studies included participants with social anxiety disorder, and 2.5% (7/275) of the studies included participants with social anxiety disorder and healthy controls. In addition, of the 275 studies, 46 (16.7%) included participants with other psychological disorders, and 34 (12.4%) included participants with other psychological disorders and healthy controls. Next, 5.1% (14/275) of the studies included participants who used substances, for example, participants who were heavy drinkers, smoked, used cannabis, used other drugs, or were recovering from substance abuse disorder.

### Question 1: Which Characteristics of the Social Environment Are Included and on Which Time Scale?

**Figure 2:**
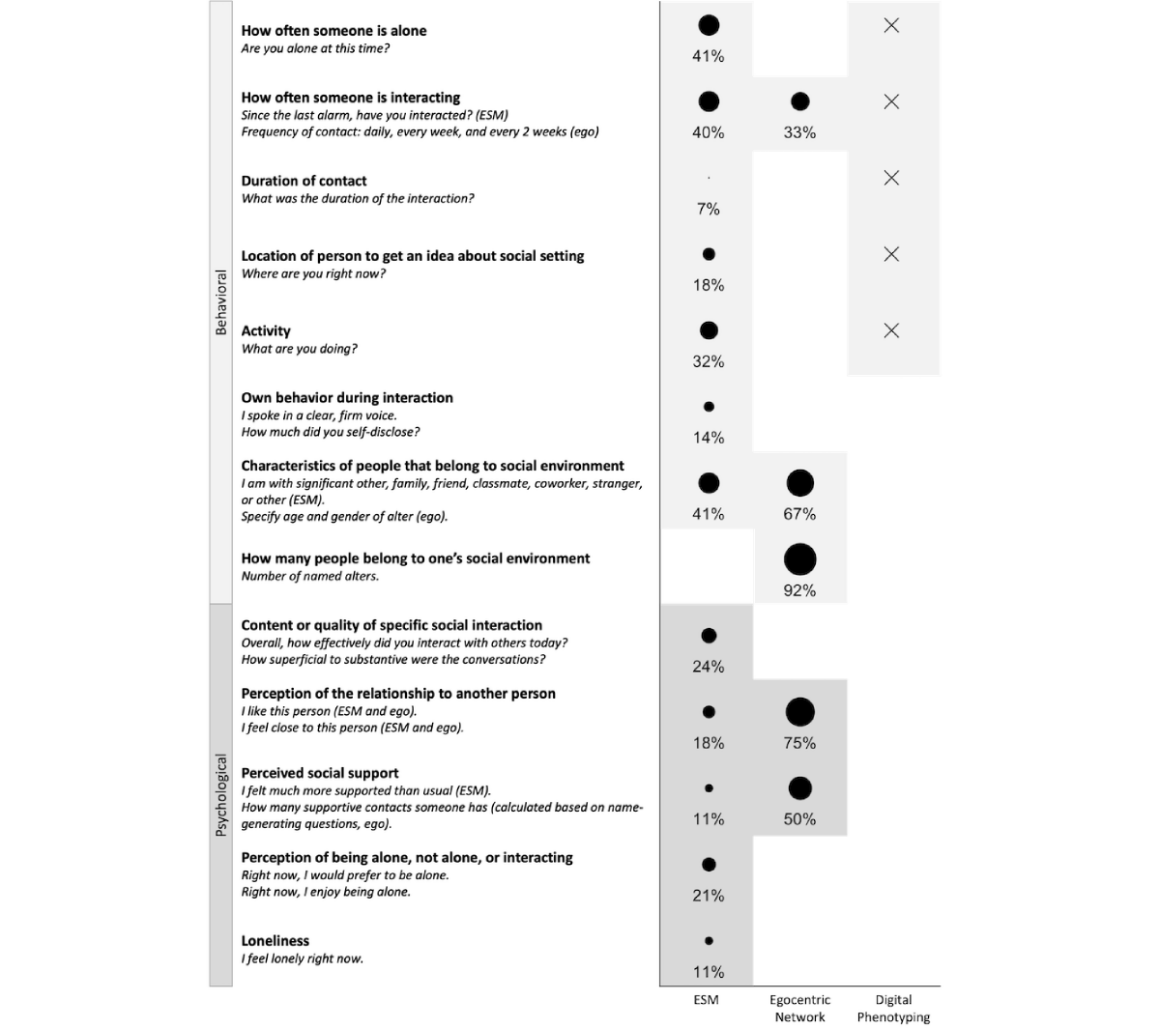
Overview of which aspects each method captures. The example items are based on the items that were used in the selected experience sampling method (ESM) and egocentric network studies. We developed categories to summarize which aspects of the social environment were captured in the selected studies. The first column shows which aspects the ESM studies captured and how often (in percentage) those aspects were measured in the 238 selected ESM studies that only used 1 measurement. Subsequently, the second column shows how often (in percentage) different aspects were included in the 12 selected egocentric network studies. The last column shows which aspects can be potentially captured using passive sensing; as those aspects are only indirectly and often implicitly measured, we did not calculate any percentages.

In this section, we describe which diverse aspects of the social environment and which temporal resolution (ie, assessment frequency and study duration) each method captured. ESM studies assessed the social environment using approximately 6 questionnaires per day, for a study length of 15 days. Questions that were asked in ESM studies were usually about what the participant was doing or feeling at the moment or since the last questionnaire. A known strength of ESM studies is that they tap into several aspects of the changing social environment by capturing both behavioral and psychological characteristics directly through questionnaires (Figure 2). The behavioral level includes what people do and the structural aspects of the social network (eg, network size). In contrast, the psychological level shows, for instance, what people feel or think [28,29]. In total, 83.6% (199/238) of the selected ESM studies included behavioral aspects, and only 66.8% (159/238) of the ESM studies included psychological aspects.

Behavioral aspects that were frequently addressed were whether someone was alone (97/238, 40.8%) or interacting (94/238, 39.5%) [43] and the characteristics of social interaction partners (97/238, 40.8%). There was less consistency across ESM studies in which psychological aspects were captured. For example, studies occasionally included questions about the content or quality of an interaction (58/238, 24.4%) [43,44] or whether someone enjoyed being alone or not alone (49/238, 20.6%) [45]. In addition, questions about relationship characteristics were occasionally asked (42/238, 17.6%) [46]. Thus, ESM studies frequently involved whether someone was not alone or interacting, but such studies were less consistent in assessing the psychological level of the social environment and social behavior.

Similar to ESM studies, a strength of egocentric network studies is that they assess behavioral (12/12, 100% of egocentric network studies) and psychological (10/12, 83% of egocentric network studies) aspects of the social environment. ESM and egocentric networks capture similar aspects of the social environment. At the behavioral level, the egocentric network studies measured characteristics of social contacts (eg, age or gender; 8/12, 67%) and occasionally asked how often someone was interacting (4/12, 33%; Figure 2), for example, by asking about the frequency of contact in the past [47]. At a psychological level, subjective relationship characteristics (9/12, 75%) and perceived social support (6/12, 50%) were often captured.

It is evident that, contrary to ESM studies, egocentric network studies focused more on the interaction partners than on the interaction itself. Studies were longer (approximately 4 years), with 3 rounds of measurements in these periods. Questions that were asked in egocentric network studies also referred to longer periods. For example, Francis [23] asked participants to name the people they had had contact with in the previous 6 months. Egocentric network studies asked a so-called name-generating question (eg, name people participants considered meaningful [47]) at the beginning of the study. This allowed them to give an estimate of the network size of a person. Furthermore, detailed information on interaction partners was available. In contrast, ESM often measures the characteristics of the social environment at a specific moment without linking them to a specific person. Even if a study specified with whom someone was interacting, often names or initials were missing and only broad categories were assessed (eg, significant other, family, friend, classmate, coworker, stranger, and other [46]).

In contrast to ESM and egocentric networks, a strength of studies that use passive sensing or EAR is that they can objectively and directly tap into behavioral aspects. These can be measured continuously using sensors during the day. The reviewed studies were, on average, 27 days long. Studies that used passive sensing or EAR mostly included the audio (microphone; 12/20, 60%) and sociability (calls or SMS text messages; 9/20, 45%) of a person. These measures can indirectly capture whether someone is alone or interacting [14,48]. In addition, mobility measures (GPS and accelerometer) were included in half (10/20, 50%) of the studies. Measures that were less often included were phone-related properties such as the total time spent on communication apps (3/20, 15%) [49].

Thus, ESM and passive sensing can both capture the location, activity, and contact frequency of a person (Figure 2). A weakness of passive sensing is that it can only indirectly capture the psychological level using its sensors (raw measures). For example, audio data must be manually coded to examine the emotions of a person [14], or passive measures must be combined with questionnaires to build models that can predict psychological variables such as loneliness [50,51]. In contrast, ESM can examine the psychological level directly by asking the person about their experiences and feelings.

### Question 2: How Well Are the Measures of the Social Environment Validated?

In the previous section, we identified which elements of the social environment were captured by the different methods. In this section, we discuss the validity of these methods. In its broadest sense, validity refers to the extent to which a method measures what it is intended to measure [52,53]. This section discusses the strategies that the selected studies chose to provide evidence of validity.

For ESM (and all other questionnaire-based measures), it is essential that items that claim to measure a specific construct do so. Obviously, this is difficult as researchers may define their constructs differently and may have different opinions about what loneliness is, for example. A way to examine the validity of the selected studies is to investigate whether validated questionnaires were used. Of the selected ESM studies, only 27.7% (66/238) used a validated questionnaire. For example, the University of California, Los Angeles, Loneliness Scale was often used to assess loneliness [54,55]. In contrast, other studies used only 1 nonvalidated question, such as “At this moment, how lonely do you feel?” [56], to assess loneliness.

Some studies (45/238, 18.9%) reported that they included items that were already used in previous research. Using items that were used in previous research does not indicate validity. However, it does make the studies more comparable with each other. Slightly more than half (126/238, 52.9%) of the studies did not use validated scales or items from previous research. Nevertheless, some authors (25/238, 10.5%) provided arguments and details about their item selection [45,57]. Overall, 42.4% (101/238) of the ESM studies did not use validated items to measure the social environment and did not give a specific reason for including the items in their research.

Questions about the behavioral level of the social environment may require less validation or explanation than questions measuring the psychological level. For example, whether someone is alone or not is easier to measure than the feeling of loneliness. Thus, it is even more important to validate questions at the psychological level. To investigate the validity of questions at the psychological level, we looked at a subset of ESM studies that included at least one question at the psychological level of the social environment (159/238, 66.8%). This subset used slightly more validation strategies (103/159, 64.8%) compared with the full sample (137/238, 57.6%).

In contrast to ESM studies, egocentric network studies aim to capture a part of the social network of a person. Validity in this case is different from that in ESM studies as the overall goal is to obtain an accurate representation of the overall network compared with measuring specific constructs. A topic that hinders validity in this regard is the size of the assessed egocentric network [24]. Depending on the number of alters included, the validity of network characteristics might differ as including too few alters might bias the network composition and structure estimates [24,26,58]. McCarty et al [26] found that naming 25 alters can capture most of the structural patterns of a network. The number of alters that participants were allowed to name differed across the included studies. In half (6/12, 50%) of the studies, participants were allowed to name an unlimited number of alters. In other studies (5/12, 42%), the number of alters that participants could include was limited and, therefore, might have biased the network estimates. However, in all except 8% (1/12) of the studies, participants were allowed to name at least 25 alters and named, on average, fewer alters than they were allowed to [59,60]. Thus, it is likely that those studies still provided valid estimates of the network structure as the limit of alters that participants were allowed to enter was not reached and at least 25 alters were allowed to be included in the network. It is important to note that how many alters participants included in their network might also be influenced by their motivation. Low motivation can lead to a biased network estimate even if unlimited alters can be added [27].

Another aspect that might affect validity in egocentric network studies is whether an appropriate name generator was used and whether the questions asked to assess the relationship characteristics were valid. Using different name generators can affect the network size and average tie characteristics [61]. Therefore, using name generators that have been used and tested in previous research might increase the validity and comparability across studies. In most studies (10/12, 83% of the egocentric network studies), the name generator questions, as well as the content questions asked to assess the relationship characteristics, had been used in previous studies. A total of 8% (1/12) of the studies did not use questions based on previous studies but only asked whom a person had contacted in the last 6 months and several characteristics of the alter [23]. A total of 8% (1/12) of the studies also did not use questions from previous research but developed their own questions based on previous literature reviews and results from earlier assessed egocentric networks [62]. Thus, except for 8% (1/12) of the studies, egocentric network studies used strategies that increased the validity and comparability across studies.

For passive sensing, validation is rather different than for questionnaire-based items, as in ESM and egocentric networks. For passive sensing, it is important that the extracted features or algorithms applied indeed capture the parts of the social environment that they are designed to capture. First, we discuss passive sensing studies excluding EAR studies. In the selected studies, only half (6/12, 50%) of the studies that only used passive sensing measured how the extracted features or the built model with a combination of features related to the changing social environment. For example, Jacobson et al [63] aimed to detect how passively collected data (ie, accelerometers and incoming and outgoing calls and SMS text messages) can predict the level of anxiety in social situations (social anxiety). They found a correlation of *r*=0.7 between predicted social anxiety severity and social anxiety measured using a questionnaire. Thus, they concluded that passive sensing can be used to detect social anxiety. We discuss this issue further in the following section on the combination of methods. The other half (6/12, 50%) of the studies that used only passive sensing (excluding EAR studies) did not mention any techniques to show how their passive collected data related to the social environment. For instance, Tsapeli and Musolesi [64] used GPS and accelerometer data as implicit indicators of social interactions. However, it remains unclear how well GPS and accelerometer data can capture social interactions. In addition, Schuwerk et al [65] used the number of contacts as an indicator of social network size [65], but it is uncertain how accurately the number of contacts can describe the social network size as no research was done to investigate this.

In EAR studies, validity is about the coding of the audio files. For example, extracted audio features from the microphone should refer to the social interactions a person had. If the coding is performed manually, this should be done in a standardized way. Predefined coding schemes can help with this process. In total, 50% (4/8) of the EAR studies used such a predefined coding scheme [14] (eg, social environment coding of sound inventory [19]). A total of 25% (2/8) of the studies used interrater reliability, meaning that the extent of agreement across different coders was measured. In 12% (1/8) of the studies, 28% of the participants listened to parts of their audio to verify the given coding [66]. In total, 12% (1/8) of the studies only looked at the presence of speech or presence of others without any specific validation [67]. In this study, this was done automatically with the Google Cloud Speech-to-Text software [67]. If the software generated a transcript, the presence of speech was assumed without active verification by a human.

Overall, ESM, egocentric networks, and passive sensing use different methods to improve the validity of their collected data. Most of the egocentric network studies (10/12, 83%) and EAR studies (7/8, 88%) included procedures that improved the validity of their collected data. In contrast, this percentage was much lower in ESM and passive sensing studies, with only approximately 60% (137/238, 57.6%) of the ESM studies and 50% (6/12) of the passive sensing studies mentioning techniques that supported the validity of their collected data.

### Question 3: How Were the Measures Combined in Previous Research?

#### Overview

To better understand how methods can be best combined, we identified 7.6% (21/275) of the studies that used more than one of our discussed methods. In total, 29% (6/21) of these studies used 1 method to assess the social environment, whereas the second method was used to capture something different (eg, mood) [68-71]. As we focus in this section on studies that used multiple methods to capture the social environment, we only discuss the remaining 71% (15/21) of the studies in more detail. We start by discussing studies that combined methods to measure several aspects of the social environment and their relation to a third variable (eg, well-being). These studies attempted to capture multiple aspects of the social environment through a combination of methods. Next, we discuss studies that examined the overlap in how different methods characterize the social environment. These studies relate to the validation of measures as different methods measured similar parts of the social environment and many studies investigated how those measures of the social environment agreed with each other.

#### Studies That Combined Different Methods to Cover a Wider Part of the Social Environment

In 20% (3/15) of the studies, different measures were combined to cover a wider part of the social environment [65,72,73]. A total of 67% (2/3) of these studies did this to investigate questions regarding different parts of the social environment. Schuwerk et al [65] investigated offline social interactions measured via ESM and interactions measured via smartphones (eg, number and duration of calls). Abel et al [72] aimed to capture social interactions objectively via EAR and, in addition, used daily questionnaires to capture subjective emotions during a social interaction [72]. Thus, a combination of methods helped answer several research questions.

Another study aimed to predict stress and mental health and highlighted that a combination of measures of the social environment (ie, passive sensing and ESM) provided better predictions of self-reported stress and poor mental health than each method on its own [73]. In this study, data were passively collected using smartphones and wearable devices. In addition, participants had to fill out a diary. Overall, using passively collected data improved the prediction of stress and mental health, although the improvements were not large (from 72% to 82% for stress and from 85% to 87% for mental health).

Dynamically assessed egocentric networks were not combined with other methods, but one-time–assessed egocentric networks were used to collect more information on interaction partners or the total network size. These studies helped better understand how repeated egocentric networks could be combined with ESM or passive sensing in future research. First, 27% (4/15) of the studies combined one-time–assessed egocentric networks with ESM to identify important contacts before the ESM data collection started [74,75], determine the relationship characteristics of daily contacts [76], or have a measure for the network size of a person [77]. It was shown that the presence of people who provide emotional support, which was measured using a combination of ESM and egocentric networks, was associated with well-being measured via ESM [76]. In addition, controlling for network size was important for understanding stress and affect [77]. Second, 13% (2/15) of the studies used (measures similar to) one-time–assessed egocentric networks together with passive sensing [50,78] to assess network members (eg, close friends, family members, and friends on campus) and determine whether someone interacted with a close friend based on Bluetooth connections or phone numbers. Hence, combining egocentric networks with passive sensing can be useful to create meaningful variables based on the passive sensing measures, such as how many close friends someone had contact with instead of just counting Bluetooth connections.

Overall, few studies combined different techniques to measure the social environment. However, combining different techniques improved the prediction and understanding of the social environment in relation to mental well-being.

#### Studies That Examined the Overlap in How the Social Environment Is Characterized by Different Methods

We identified 27% (4/15) of studies that indicated that passively collected data correlated with parts of the social environment and social behavior assessed using ESM [79-82]. First, Abdullah et al [79] found promising results using passive measures such as nonsedentary duration based on the accelerometer and conversation frequency based on the microphone to predict how stable the social rhythm of a person is. The social rhythm of a person was measured through ESM questions on the daily routine, such as at what time someone would get out of bed and have their first social contact. In addition, other studies found a relationship between passive measures (such as total time traveled based on GPS) and a person’s social context and activity (such as whether a person was alone, having a conversation, interacting, or in a location [80-82]). However, the strength of the association differed by study, measure, and participant group. For example, Fulford et al [81] showed that passive measures related to an individual’s social behavior differed for individuals with schizophrenia and healthy controls. The association between distance traveled and number of interactions ranged from 0.07 for people with schizophrenia to 0.6 for healthy controls [81]. In general, they concluded that passively collected mobility features (GPS) relate moderately to ESM measures of social behavior for healthy individuals (ranging from a correlation of *r*=0.31 to *r*=0.7), whereas audio relates moderately to ESM measures of social behavior for people with schizophrenia (correlations of approximately 0.5). Overall, this 27% (4/15) of studies indicated that there are moderate associations between passive measures and social behavior but that it is difficult to draw any final conclusions because of the variation in methods and results across and within studies.

In the remainder of this section, we describe 13% (2/15) of studies that used EAR together with ESM. Interestingly, studies that used ESM together with EAR only showed moderate agreement between the assessed variables. Minor et al [21] investigated how EAR is implemented in students with schizotypy and how social engagement differs between students characterized by high or low schizotypy. Students had to wear EAR for 2 days and fill out ESM for 16 days. Surprisingly, even though the conclusions drawn from each measurement were similar, there was little overlap between EAR and ESM measures for positive and negative affect. The authors argue that both measures capture different facets of daily life. EAR captures affect without the interpretation of the participant, whereas ESM adds a subjective context to it, such as the quality of the relationship between interaction partners. Similarly, Sun et al [51] used EAR and ESM to measure the relationship between the quantity and quality of social interaction and well-being. They only found a moderate agreement between EAR and ESM regarding when participants were interacting (*r*=0.39), conversational depth (*r*=0.31), and self-disclosure during an interaction (*r*=0.31).

To conclude, our results indicate that passively collected data and ESM assess overlapping aspects of the social environment but that more validation studies are needed to investigate which aspects are robustly measured with passive sensing data across studies [79-81]. In addition, studies that used ESM together with EAR only showed moderate agreement between the assessed variables. This indicates that the convergent validity between the 2 measures is not high, meaning that different measures of the same construct do not agree much [83]. Nevertheless, the overall conclusions drawn from both measures (ESM and EAR) regarding the relationship to schizotypy and well-being were similar [21,51].

## Discussion

### Principal Findings

Our aim was to study how distinct methods originating from different disciplines were used to capture people’s changing social environments, identify the strengths and weaknesses of each method, and detect opportunities to combine them. We focused on how ESM, passive sensing, and repeated egocentric networks are currently used to measure the social environment. We investigated how parts of the social environment were captured by each method and the validity of the measures used in each method. Furthermore, we examined how these methods have been combined in previous research.

In total, 3 major implications follow from our literature review, which we will discuss in detail in the following sections, and are summarized in Textbox 1. First, a combination of methods is essential to better capture the changing social environment as each method captures a different resolution (duration and frequency) of the social environment and assesses aspects of the social environment at different levels (psychological vs behavioral). However, a combination of methods has rarely been observed in previous research. Second, a combination of methods has tremendous potential to reduce both researcher and participant burden as there is overlap in the various constructs they capture. Third, measures of the social environment are rarely validated, which undermines the robustness of the conclusions drawn. We will provide suggestions in the following sections for future efforts to collect and analyze data.

**Recommendations for future studies measuring the changing social environment.**

***Combine methods to achieve a more comprehensive picture of the social environment***

- Tap into more aspects of the social environment by using different methods to capture what people do and what people feel and perceive. Frequently assessed aspects in the included studies were the following:
  What people do: how often someone is alone, how often someone is interacting, duration of an interaction, location, activity, own behavior during interaction (measured using the experience sampling method [ESM]), characteristics of persons that belong to the social environment, and how many people belong to one’s social environment (network size, measured using egocentric networks)
  What people think and perceive: content or quality of a specific interaction (measured using ESM), subjective perception of the relationship to another person, perceived social support, perception of being alone, not alone, or interacting (measured using ESM), and loneliness (measured using ESM)

***Combine methods to measure aspects on a suitable frequency and reduce researcher and participant burden***

- Choose a suitable time scale for capturing the constructs of interest, which means that fluctuating aspects should be measured using ESM and passive sensing and stable aspects can be captured using repeated egocentric networks (make sure to collect identifiers [nicknames or initials] in the ESM to collect the egocentric network). Stable characteristics might be the following:
  Characteristics of a person that belongs to one’s social network (eg, gender; age; and objective relationship characteristics such as friend, colleague, or coworker)
  Subjective relationship characteristics that do not change daily (eg, how close someone is)
  Perceived social support to specific persons
- Consider using passive measures to reduce the length of ESM questionnaires. For example, location or physical activity can potentially be assessed using passive sensing.

***Better validation for ESM and passive sensing***

- Use validated ESM items or other strategies that increase validity. Other strategies that were used in the studies in our review were the following:
  Choosing questions based on previous research (eg, ESM Item Repository)
  Providing arguments for why a specific item was included (especially if it was not validated)
  Choosing ESM items that relate to validated cross-sectional scales
  Explanation of the ESM questions to the participants
  Doing a test round or pilot round
  Doing a multiverse analysis
  Choosing a suitable method for a psychometric evaluation of the items
- Make explicit what one aims to measure using passive measures (ie, which part of the changing social environment one aims to capture).
- To validate which part of the changing social environment the passive measures capture, consider using ESM in addition.

### Combining Methods Will Achieve a More Comprehensive Picture of the Social Environment and Can Reduce Participant Burden (Compared With Only Using ESM)

We argue that future research would benefit from combining different methods to capture a wider part of the social environment. A combination will help measure aspects that would have been missed by a single method but are important for well-being. The results from Sano et al [73] substantiate this conclusion by showing that combining ESM and passive sensing provides better well-being predictions than each method on its own. However, our results show that a combination of methods has rarely been observed in previous research. Only 5.5% (15/275) of the studies combined ESM and passive sensing, and none of the selected studies combined all 3 methods.

Importantly, it is well known that ESM is suited to capture both the behavioral and psychological aspects of the social environment and, therefore, is a great tool for capturing a wide part of the social environment. However, our results showed that, even though ESM can capture both levels, approximately one-third of the ESM studies (79/238, 33.2%) only captured the behavioral level (eg, whether someone was alone or interacting) without assessing the psychological level (eg, how the quality of the interaction was perceived). This does not align with previous research that showed that the psychological aspects of the social environment are important to understand well-being [7,46]. Thus, we would recommend that future studies include more aspects of the psychological level as ESM studies are especially suited to measure in-the-moment experiences.

The remainder of this section focuses on how these methods can be combined. First, combining ESM with egocentric networks would be beneficial to measure the variable of interest at a suitable frequency. On the one hand, it is known and supported by our results that egocentric networks are often only assessed infrequently (eg, twice a year). However, substantive fluctuations in mood and behavior occur over shorter time scales, and ESM is needed to capture those fluctuations [11]. In contrast, ESM measures the social environment several times a day, but some relationship characteristics might be more stable. Thus, similarly to Hopwood et al [84], we recommend explicitly thinking about the duration and frequency of change of a (social) construct and determining how often and frequently it should be measured based on a theoretical conceptualization [84]. We recommend capturing variables that do not change frequently using egocentric networks instead of using ESM. This reduces the length of daily ESM questionnaires and, thus, the participant burden [85].

For example, we know that whether a contact is close or not or the characteristics of an interaction partner (eg, gender) do not change frequently during the day. Nevertheless, our results show that these questions, if asked in ESM studies [46], are often asked multiple times a day. In the reviewed studies, often the identities of interaction partners were missing, and only general categories were assessed (eg, significant other, family, friend, classmate, coworker, stranger, and other [46]). Thus, our results indicate that the same questions on interaction partners may be asked multiple times per day. This is contrary to the purpose of ESM, which is designed to measure in-the-moment experiences rather than static characteristics. Hence, for future research, it would be beneficial to assess the names or initials of an interaction partner in ESM studies and measure more static relationship characteristics using an egocentric network at the beginning or end of a study (such as the nature of the relationship, closeness, and characteristics of the relationship with the interaction partner).

Some of the selected studies already did something similar by assessing the close contacts of a person using a questionnaire before passive sensing or ESM started [50,75,76]. This information helped cover more aspects of the social environment and shorten the ESM questionnaire. Moreover, Sun et al [51] proposed to assess relationship characteristics using a baseline questionnaire and only ask people during ESM who they had interacted with based on this questionnaire. Currently, Stadel [86] is working on combining ESM and (one-time–assessed) egocentric networks for clinical practice.

Passive sensing measures might be further useful to reduce the length of ESM questionnaires. Our review identified that some aspects of the social environment are captured via passive sensing and ESM, such as a person’s location and activities [64,87]. Given the major burden for respondents to frequently report their activities and location through ESM, which reduces the validity of the data and response rate [88,89], passive sensing can be an important tool to assess the activity and location at a high resolution and for a long duration with low participant burden. There is an additional opportunity to replace ESM questions about the social environment (eg, number of interactions) with passive sensing measures (eg, distance traveled), which would further reduce participant burden [79-81,90]. However, it is too premature to make more specific recommendations as it is not yet clear how much overlap exists between passive measures and ESM and research suggests that it varies across studies and individuals. Furthermore, different methods might capture different underlying constructs even if they are supposed to measure the same ones [21,51]. Further validation is needed before particular questions can be replaced with passive sensing.

### Improving Validation Techniques

Although the included studies attempted to characterize the social environment, we identified shortcomings in the use of validated measures to capture the social environment. We observed two main problems with respect to the validity of the data: (1) the use of nonvalidated items in ESM studies and (2) the use of features in passive sensing studies related to the social environment that were not validated.

Approximately 70% (172/238, 72.3%) of the ESM studies did not use items that were based on validated questionnaires. This is in line with recent articles emphasizing that psychological research has a major issue with validation and questionnaires without validation are applied frequently [33,91,92]. Using nonvalidated questionnaires can lead to problems in the interpretation of research results and reduce the robustness of the study conclusions [33]. It further makes it hard to compare results across studies.

A way forward to improve ESM measures would be to conduct more studies that validate measures for different aspects of the social environment and use strategies that improve the validity of the measures. For example, as described by Mestdagh and Dejonckheere [91], recent innovations such as the ESM Item Repository [93] or multiverse analysis [94] are a first step toward improving the reliability and validity of ESM item selection. Fortunately, different statistical methods have also become available that can be used to evaluate the reliability of ESM items [92,95-99].

In contrast to ESM, validity in egocentric network studies refers to obtaining an accurate estimate of network characteristics. This estimate highly depends on the number of alters included [24,26,58]. In addition, using an appropriate name generator and questions to assess the relationship characteristics is important. Half (6/12, 50%) of the selected studies allowed the participants to name an unlimited number of alters. Even though the number of alters was limited in some studies, participants named, on average, less alters than they were allowed to. Thus, it is likely that these studies still provided a valid estimate of the network characteristics. The name generator and questions asked to assess the relationship characteristics were mostly used in previous research, which increases comparability (>80%).

Passive sensing suffers from a lack of studies that show that features based on passively collected data relate to the construct that is intended to be measured. Although some studies (6/12, 50%) used pre- and postassessment questionnaires [63] to investigate how passive measures related to the social environment, 50% (6/12) of the studies that only used passive sensing did not use any validation to show how their passive measures relate to the social behavior of a person (or other intended measures). For example, GPS and accelerometer data were used as implicit indicators of social interactions [64], and the number of contacts was used as an indicator of social network size [65]. It is entirely unclear how well these features map to what they are supposed to measure.

In addition, the studies were often vague about which social behavior they aimed to capture with the use of passive measures. They often divided their features into “mobility” and “social” features and did not explicitly indicate which part of the social environment passive sensors aimed to capture [48,100]. Depending on the research question, this might not be a problem, for example, if the aim is mainly to predict particular outcomes. However, it hinders the understanding of which social mechanism shapes well-being when it is unclear which behavior is captured using passive sensing. Thus, we propose clearly defining which constructs passive measures aim to capture and validating those measures.

Some of the studies that already combined different measures using ESM and passive sensing together showed that there is overlap between the features created through passive sensing and the variables assessed via ESM, which is another way to validate passive measures. However, these studies found that features differ across studies and individuals (eg, people with schizophrenia vs healthy controls [81]). Thus, we cannot draw any final conclusions about which ESM questionnaires are captured using passive sensing. For future research that aims to use passive sensing to capture the changing social environment, it is important to investigate whether passive sensing can be used to capture aspects of the social environment other than call and SMS text message behavior and, if so, to identify which features can be used.

### Limitations

Although there are several strengths to this review, including its contribution to describing a way forward for how the social environment can be measured from a holistic perspective and the inclusion of many studies from multiple databases, some limitations warrant discussion. A limitation was that only 1 researcher screened the papers. Thus, especially the coding of which aspects of the social environment were captured using each method was not checked for interrater reliability. Nevertheless, the categories were created as an iterative process, and specific cases were discussed with all the researchers. We described each category and chose example items to increase the objective categorization of ESM questions, egocentric network questions, and passive sensors. Given that our interest lies in broadly describing the use of different methods to capture the social environment, minor changes in coding would not likely affect our general conclusions.

Regarding passive sensing, there were several studies that did not aim to measure the social environment but to investigate more generally how passive measures relate to well-being. Our review only included studies that explicitly aimed to use passive measures that related to the broader social environment of a person. Thus, studies that captured parts of the social environment using passive measures but did not explicitly mention this were not included in this review. These studies can be found in systematic reviews that cover the relationship between passive sensors and well-being more generally [36,37,101,102]. Recently, a new method has also evolved (ie, screenomics [103]) that captures the digital behavior of a person on their smartphone passively via multiple screenshots. As this method mainly focuses on the digital environment of a person, it was not included in our review, which aimed to capture the broad social environment of a person.

In our scoping review, we focused on studies that measured the social environment in relation to well-being in daily life. Thus, we excluded studies that measured the social environment only once or that primarily focused on medical conditions (eg, stroke) or only on dyadic relationships. We also excluded studies that only focused on children and adolescents or older people. For future research, it might be interesting to look at such medical studies and studies with children and adolescents or older people and investigate how they measured the social environment. In these groups, the social environment is similarly important for well-being.

### Conclusions

Social interactions are important for well-being, which is widely recognized by researchers from different fields. Many different disciplines have developed measures that are able to capture changes in the social environment. In this study, we synthesized the literature on how different measures were used to capture the dynamic social environment in relation to well-being. Our results indicate that the combination of measures is currently lacking but that a combination is important to capture the social environment from a holistic perspective. Therefore, we propose combining these methods more often to reduce researcher and participant burden to improve data quality and cover more aspects of the changing social environment, which is needed to better predict a change in well-being. Finally, we call for more research that validates the measures used to capture aspects of the social environment.

## Appendix

Multimedia Appendix 1 PRISMA (Preferred Reporting Items for Systematic Reviews and Meta-Analyses) 2020 checklist.

Multimedia Appendix 2 Literature search strategy.

Multimedia Appendix 3 Overview of included studies.

## Abbreviations

EAR: electronically activated recorder
ESM: experience sampling method
PRISMA: Preferred Reporting Items for Systematic Reviews and Meta-Analyses
