# Supplementary material for: Capturing the Dynamics of the Social Environment Through Experience Sampling Methods, Passive Sensing, and Egocentric Networks: Scoping Review
Source: JMIR Ment Health. 2023 Mar 17;10:e42646. doi: 10.2196/42646 (PMC10132048; doi:10.2196/42646)
Supplement: Multimedia Appendix 1 [file mental_v10i1e42646_app1.docx]

| **Section and Topic** | **Item #** | **Checklist item** | **Location where item is reported** |
| --- | --- | --- | --- |
| **TITLE** | | |  |
| Title | 1 | Identify the report as a systematic review. | Title (Since we conducted a scoping review our title is: “Capturing the Dynamics of the Social Environment through Experience Sampling Methods, Digital Phenotyping, and Egocentric Networks - a Scoping Review”. |
| **ABSTRACT** | | |  |
| Abstract | 2 | See the PRISMA 2020 for Abstracts checklist. | *Done (Due to limited words, we did not specify the eligibility criteria, information source, risk of bias, limitations, funding, and registration in our abstract).* |
| **INTRODUCTION** | | |  |
| Rationale | 3 | Describe the rationale for the review in the context of existing knowledge. | Introduction |
| Objectives | 4 | Provide an explicit statement of the objective(s) or question(s) the review addresses. | Introduction |
| **METHODS** | | |  |
| Eligibility criteria | 5 | Specify the inclusion and exclusion criteria for the review and how studies were grouped for the syntheses. | Method: Eligibility Criteria. *No grouping for syntheses.* |
| Information sources | 6 | Specify all databases, registers, websites, organisations, reference lists and other sources searched or consulted to identify studies. Specify the date when each source was last searched or consulted. | Method: Literature Search Strategy |
| Search strategy | 7 | Present the full search strategies for all databases, registers and websites, including any filters and limits used. | Method: Literature Search Strategy & Supplementary Material (SA.1) |
| Selection process | 8 | Specify the methods used to decide whether a study met the inclusion criteria of the review, including how many reviewers screened each record and each report retrieved, whether they worked independently, and if applicable, details of automation tools used in the process. | Method: Eligibility Criteria. |
| Data collection process | 9 | Specify the methods used to collect data from reports, including how many reviewers collected data from each report, whether they worked independently, any processes for obtaining or confirming data from study investigators, and if applicable, details of automation tools used in the process. | Method: Data Extraction |
| Data items | 10a | List and define all outcomes for which data were sought. Specify whether all results that were compatible with each outcome domain in each study were sought (e.g. for all measures, time points, analyses), and if not, the methods used to decide which results to collect. | Method: Data Extraction |
|  | 10b | List and define all other variables for which data were sought (e.g. participant and intervention characteristics, funding sources). Describe any assumptions made about any missing or unclear information. | Method: Data Extraction |
| Study risk of bias assessment | 11 | Specify the methods used to assess risk of bias in the included studies, including details of the tool(s) used, how many reviewers assessed each study and whether they worked independently, and if applicable, details of automation tools used in the process. | Method: Risk of Bias |
| Effect measures | 12 | Specify for each outcome the effect measure(s) (e.g. risk ratio, mean difference) used in the synthesis or presentation of results. | *No effect measures were used.* |
| Synthesis methods | 13a | Describe the processes used to decide which studies were eligible for each synthesis (e.g. tabulating the study intervention characteristics and comparing against the planned groups for each synthesis (item #5)). | *Since we did a narrative analysis, this is not applicable.* |
|  | 13b | Describe any methods required to prepare the data for presentation or synthesis, such as handling of missing summary statistics, or data conversions. | Method: Eligibility Criteria |
|  | 13c | Describe any methods used to tabulate or visually display results of individual studies and syntheses. | *Since we did a narrative analysis, this is not applicable.* |
|  | 13d | Describe any methods used to synthesize results and provide a rationale for the choice(s). If meta-analysis was performed, describe the model(s), method(s) to identify the presence and extent of statistical heterogeneity, and software package(s) used. | *As stated in the abstract, we use a narrative synthesis based on the four research questions that are stated in the Introduction* |
|  | 13e | Describe any methods used to explore possible causes of heterogeneity among study results (e.g. subgroup analysis, meta-regression). | *Since we did a narrative analysis focused on the methods this is not applicable.* |
|  | 13f | Describe any sensitivity analyses conducted to assess robustness of the synthesized results. | *Since we did a narrative analysis focused on the methods no sensitivity analysis was conducted.* |
| Reporting bias assessment | 14 | Describe any methods used to assess risk of bias due to missing results in a synthesis (arising from reporting biases). | *Our primary aim was to identify studies that used measures to capture the changing social environment. Thus, the outcome and quality of studies were less relevant for this review and would not have impacted the eligibility of studies. Therefore, the risk of bias for each study was not assessed.* |
| Certainty assessment | 15 | Describe any methods used to assess certainty (or confidence) in the body of evidence for an outcome. | *Since we did a narrative analysis focused on the methods certainty was not assessed.* |
| **RESULTS** | | |  |
| Study selection | 16a | Describe the results of the search and selection process, from the number of records identified in the search to the number of studies included in the review, ideally using a flow diagram. | Results: Overview of Selected Studies, Figure 1 & Figure 2 |
|  | 16b | Cite studies that might appear to meet the inclusion criteria, but which were excluded, and explain why they were excluded. | Supplementary Material (SB.1-3) |
| Study characteristics | 17 | Cite each included study and present its characteristics. | Supplementary Material (SB. 4) |
| Risk of bias in studies | 18 | Present assessments of risk of bias for each included study. | *Our primary aim was to identify studies that used measures to capture the changing social environment. Thus, the outcome and quality of studies were less relevant for this review and would not have impacted the eligibility of studies. Therefore, the risk of bias for each study was not assessed.* |
| Results of individual studies | 19 | For all outcomes, present, for each study: (a) summary statistics for each group (where appropriate) and (b) an effect estimate and its precision (e.g. confidence/credible interval), ideally using structured tables or plots. | *Since we did a narrative analysis focused on the methods this is not applicable.* |
| Results of syntheses | 20a | For each synthesis, briefly summarise the characteristics and risk of bias among contributing studies. | *Our primary aim was to identify studies that used measures to capture the changing social environment. Thus, the outcome and quality of studies were less relevant for this review and would not have impacted the eligibility of studies. Therefore, the risk of bias for each study was not assessed.* |
|  | 20b | Present results of all statistical syntheses conducted. If meta-analysis was done, present for each the summary estimate and its precision (e.g. confidence/credible interval) and measures of statistical heterogeneity. If comparing groups, describe the direction of the effect. | *Since we did a narrative analysis focused on the methods no statistical analysis (besides basic descriptives) was done.* |
|  | 20c | Present results of all investigations of possible causes of heterogeneity among study results. | *Not applicable* |
|  | 20d | Present results of all sensitivity analyses conducted to assess the robustness of the synthesized results. | *No sensitivity analyses done (narrative review)* |
| Reporting biases | 21 | Present assessments of risk of bias due to missing results (arising from reporting biases) for each synthesis assessed. | *Not applicable (narrative review)* |
| Certainty of evidence | 22 | Present assessments of certainty (or confidence) in the body of evidence for each outcome assessed. | *Not applicable (narrative review)* |
| **DISCUSSION** | | |  |
| Discussion | 23a | Provide a general interpretation of the results in the context of other evidence. | Discussion |
|  | 23b | Discuss any limitations of the evidence included in the review. | Discussion: Limitations |
|  | 23c | Discuss any limitations of the review processes used. | Discussion: Limitations |
|  | 23d | Discuss implications of the results for practice, policy, and future research. | Discussion/ Discussion: Conclusion. *No direct implications for policy* |
| **OTHER INFORMATION** | | |  |
| Registration and protocol | 24a | Provide registration information for the review, including register name and registration number, or state that the review was not registered. | *The review was not registered* |
|  | 24b | Indicate where the review protocol can be accessed, or state that a protocol was not prepared. | *The protocol was not prepared* |
|  | 24c | Describe and explain any amendments to information provided at registration or in the protocol. | *-* |
| Support | 25 | Describe sources of financial or non-financial support for the review, and the role of the funders or sponsors in the review. | Statements and Declarations |
| Competing interests | 26 | Declare any competing interests of review authors. | Statements and Declarations |
| Availability of data, code and other materials | 27 | Report which of the following are publicly available and where they can be found: template data collection forms; data extracted from included studies; data used for all analyses; analytic code; any other materials used in the review. | Statements and Declarations |

*From:*  Page MJ, McKenzie JE, Bossuyt PM, Boutron I, Hoffmann TC, Mulrow CD, et al. The PRISMA 2020 statement: an updated guideline for reporting systematic reviews. BMJ 2021;372:n71. doi: 10.1136/bmj.n71

For more information, visit: <http://www.prisma-statement.org/>
