# Supplementary material for: Capturing the Dynamics of the Social Environment Through Experience Sampling Methods, Passive Sensing, and Egocentric Networks: Scoping Review
Source: JMIR Ment Health. 2023 Mar 17;10:e42646. doi: 10.2196/42646 (PMC10132048; doi:10.2196/42646)
Supplement: Multimedia Appendix 2 [file mental_v10i1e42646_app2.docx]

**Supplementary Material A - Capturing the Dynamics of the Social Environment - A Systematic Review on Methodologies**

**SA.1) Literature Search Strategy**

We used two different search strings. The first search string aimed to identify studies that measured the social environment with ESM or digital phenotyping or a combination of methods. The second search string aimed to identify studies that measured the social environment with repeated egocentric networks or a combination of methods.

ESM and digital phenotyping are by definition methods that capture the social environment dynamically but are also used to measure other constructs. Therefore, we used the following search string: (Social Environment) AND (ESM OR Digital Phenotyping) AND (well-being) NOT (older adults OR elderly OR child* OR MICE OR animal* OR rats). For each term, we added synonyms into the search strings. Those synonyms were mainly based on previous systematic reviews [36-38].

The second search string aimed to identify studies that measured the social environment with repeated egocentric networks or a combination of methods. Egocentric networks are generally measuring the social environment but are not always used to capture change. Thus, we used the following search string: (ego network) AND (longitudinal) NOT (older adults OR elderly OR child* OR MICE OR animal* OR rats). We added synonyms inspired by previous systematic reviews [38]. Both full search strings, including the synonyms in each database, can be found below.

***First search was done in December 2020, updated on the 05.07.21***

Web of Knowledge

*1) ESM + Digital Phenotyping*

(TS=("loneliness" OR "isolation" OR "social support" OR "emotional support" OR "social interactions" OR "social interaction" OR "social environment" OR "social context" OR "social contacts" OR "social capital" OR "social integration" OR "social influence" OR "social dynamics" OR "social participation" OR "social network*" OR "social relationship*" OR "social network*" OR "interpersonal relations" OR "social behav*" ) OR (TI =(social*) )) AND TS=("ESM" OR "experience sampling" OR "EMA" OR "Ambulatory Assessment" OR "ecological momentary" OR " Experience Sampling Methodology" OR "diary stud*" OR "ambulatory monitoring" OR "digital phenoty*" OR "behavioral phenoty*" OR "smartphone phenoty*" OR "phone phenoty*" OR "social sensing" OR "social sensor" OR "passive sensing" OR "passive sensor" OR "Smartphone Sensing" OR "smartphone sensor" OR "phone sensing" OR "phone sensor" OR "digital sensing" OR "digital sensor" OR "passive data" OR "sensor data" OR "passive behavioral monitoring" OR "smartphone monitoring" OR "phone monitoring" OR "digital monitoring" OR "passive behavioral detection" OR "smartphone detection" OR "phone detection" OR "digital detection" OR "passive behavioral detector" OR "smartphone detector" OR "phone detector" OR "digital detector" OR "passive behavioral measurement" OR "smartphone measurement" OR "phone measurement" OR "digital measurement" OR "Passive Telemetric Monitoring" OR "Ambulatory Assessment of Movement Behavior" OR "Electronically Activated Recorder" OR "EAR" OR "wearable computing" OR "smartphone based" OR "smartphone-based" OR "digital biomarkers" OR "accelerometer" OR "microphone" OR "global positioning system" ) AND TS= ("mental health" OR "wellbeing" OR "well-being" OR "positive affect" OR "negative affect" OR "mood" OR "depression" OR "stress" OR "schizophrenia" OR "anxiety" OR "relapse" OR "disorder" OR "bipolar" OR "happiness" OR "life satisfaction" OR "psychiatry" OR "psychopathology" OR "psychiatric symptom*" OR "mental state*" OR "psychological state*" OR "affective state*" OR "psychological distress") NOT TS=("older adult*" OR "elderly" OR "child*" OR "mice" OR "animal*" OR “rats”)

*2) Ego Networks*

TS=("ego network*" OR "personal network*" OR "ego-network*" OR "egocentric network"  OR "individual social network*" OR "personal social network*") AND TS=("mental health" OR "wellbeing" OR "well-being" OR "positive affect" OR "negative affect" OR "mood" OR "depression" OR "stress" OR "schizophrenia" OR "anxiety" OR "relapse" OR "disorder" OR "bipolar" OR "happiness" OR "life satisfaction" OR "psychiatry" OR "psychopathology" OR "psychiatric symptom*" OR "mental state*" OR "psychological state*" OR "affective state*" OR "psychological distress" OR "psychology" OR "mental distress") AND TS = ("longitudinal*" OR "time-series" OR "time series" OR "dynamic*" OR "repeated" OR "change*" OR "long term" OR "long-term") NOT TS = ("older adult*" OR "elderly" OR "child*" OR "mice" OR "animal*" OR “rats”)

PsychInfo

*1) ESM + Digital Phenotyping*

(“loneliness” OR “isolation” OR  “social support” OR “emotional support” OR “social interactions” OR “social interaction” OR “social environment” OR “social context” OR “social contacts” OR “social capital” OR “social integration” OR “social influence” OR “social dynamics” OR “social participation” OR “social network*” OR “social relationship*” OR “social network*” OR “interpersonal relations” OR “social behav*” OR TI = (social*)) AND (“ESM” OR “experience sampling” OR “EMA” OR “Ambulatory Assessment” OR “ecological momentary” OR “Experience Sampling Methodology” OR “diary stud*” OR “ambulatory monitoring” OR “digital phenoty*” OR “behavioral phenoty*” OR “smartphone phenoty*” OR  “phone phenoty*” OR “social sensing” OR “social sensor” OR “passive sensing” OR “passive sensor” OR “Smartphone Sensing” OR “smartphone sensor” OR “phone sensing” OR “phone sensor” OR “digital sensing” OR “digital sensor” OR “passive data”  OR “sensor data”  OR “passive behavioral monitoring” OR “smartphone monitoring” OR “phone monitoring”  OR “digital monitoring” OR “passive behavioral detection” OR “smartphone detection” OR “phone detection”  OR “digital detection”  OR  “passive behavioral detector” OR “smartphone detector” OR “phone detector”  OR “digital detector”  OR “passive behavioral measurement” OR “smartphone measurement” OR “phone measurement”  OR “digital measurement” OR  “Passive Telemetric Monitoring” OR “Ambulatory Assessment of Movement Behavior” OR “Electronically Activated Recorder” OR “EAR” OR “wearable computing” OR “smartphone based” OR “smartphone-based” OR “digital biomarkers” OR “accelerometer” OR “microphone” OR “global positioning system” ) AND (“mental health” OR “wellbeing” OR “well-being” OR “positive affect” OR “negative affect” OR “mood” OR “depression” OR “stress” OR “schizophrenia” OR “anxiety” OR “relapse” OR “disorder” OR “bipolar” OR “happiness” OR “life satisfaction” OR “psychiatry” OR “psychopathology” OR “psychiatric symptom*” OR “mental state*”  OR “psychological state*” OR “affective state*” OR “psychological distress”) NOT (“older adult*” OR “elderly” OR “child*” OR “mice” OR “animal*” OR “rats”)

*2) Ego Networks*

(“ego network*” OR “personal network*” OR “ego-network*”  OR “egocentric network” OR “individual social network*” OR “personal social network*”) AND (“mental health” OR “wellbeing” OR “well-being” OR “positive affect” OR “negative affect” OR “mood” OR “depression” OR “stress” OR “schizophrenia” OR “anxiety” OR “relapse” OR “disorder” OR “bipolar” OR “happiness” OR “life satisfaction” OR “psychiatry” OR “psychopathology” OR “psychiatric symptom*” OR “mental state*”  OR “psychological state*” OR “affective state*” OR “psychological distress” OR “psychology” OR “mental distress”) AND (“longitudinal*” OR “time-series” OR “time series” OR “dynamic*” OR “repeated” OR “change*” OR “long term” OR “long-term”) NOT (“older adult*” OR “elderly” OR “child*” OR “mice” OR “animal*” OR “rats”)

Pubmed

*1) ESM + Digital Phenotyping*

("loneliness"[Title/Abstract] OR "isolation"[Title/Abstract] OR "social support"[Title/Abstract]

 OR "emotional support"[Title/Abstract] OR "social interactions"[Title/Abstract] OR "social interaction"[Title/Abstract] OR "social environment"[Title/Abstract] OR "social context"[Title/Abstract] OR "social contacts"[Title/Abstract] OR "social capital"[Title/Abstract] OR "social integration"[Title/Abstract] OR "social influence"[Title/Abstract] OR "social dynamics"[Title/Abstract] OR "social participation"[Title/Abstract] OR "social network*"[Title/Abstract] OR "social relationship*"[Title/Abstract] OR "social network*"[Title/Abstract] OR "interpersonal relations"[Title/Abstract] OR "social behav*"[Title/Abstract] OR "social"[Title]) AND ("ESM"[Title/Abstract] OR "experience sampling"[Title/Abstract] OR "EMA"[Title/Abstract] OR "Ambulatory Assessment"[Title/Abstract] OR "ecological momentary"[Title/Abstract] OR " Experience Sampling Methodology"[Title/Abstract]

 OR "diary stud*"[Title/Abstract] OR "ambulatory monitoring"[Title/Abstract] OR "digital phenoty*"[Title/Abstract] OR "behavioral phenoty*"[Title/Abstract] OR "smartphone phenoty*"[Title/Abstract] OR "phone phenoty*"[Title/Abstract] OR "social sensing"[Title/Abstract] OR "social sensor"[Title/Abstract] OR "passive sensing"[Title/Abstract] OR "passive sensor"[Title/Abstract] OR "Smartphone Sensing"[Title/Abstract] OR "smartphone sensor"[Title/Abstract] OR "phone sensing"[Title/Abstract] OR "phone sensor"[Title/Abstract] OR "digital sensing"[Title/Abstract] OR "digital sensor"[Title/Abstract]

 OR "passive data"[Title/Abstract] OR "sensor data"[Title/Abstract] OR "passive behavioral monitoring"[Title/Abstract] OR "smartphone monitoring"[Title/Abstract] OR "phone monitoring"[Title/Abstract] OR "digital monitoring"[Title/Abstract] OR "passive behavioral detection"[Title/Abstract] OR "smartphone detection"[Title/Abstract] OR "phone detection"[Title/Abstract] OR "digital detection"[Title/Abstract] OR "passive behavioral detector"[Title/Abstract] OR "smartphone detector"[Title/Abstract] OR "phone detector"[Title/Abstract] OR "digital detector"[Title/Abstract] OR "passive behavioral measurement"[Title/Abstract] OR "smartphone measurement"[Title/Abstract] OR "phone measurement"[Title/Abstract] OR "digital measurement"[Title/Abstract] OR "Passive Telemetric Monitoring"[Title/Abstract] OR "Ambulatory Assessment of Movement Behavior"[Title/Abstract] OR "Electronically Activated Recorder"[Title/Abstract] OR "EAR"[Title/Abstract] OR "wearable computing" [Title/Abstract]OR "smartphone based"[Title/Abstract] OR "smartphone-based"[Title/Abstract] OR "digital biomarkers"[Title/Abstract] OR "accelerometer"[Title/Abstract] OR "microphone"[Title/Abstract] OR "global positioning system"[Title/Abstract]) AND ("mental health"[Title/Abstract] OR "wellbeing"[Title/Abstract] OR "well-being"[Title/Abstract] OR "positive affect"[Title/Abstract] OR "negative affect"[Title/Abstract] OR "mood"[Title/Abstract] OR "depression"[Title/Abstract] OR "stress"[Title/Abstract] OR "schizophrenia"[Title/Abstract] OR "anxiety"[Title/Abstract] OR "relapse"[Title/Abstract] OR "disorder"[Title/Abstract] OR "bipolar"[Title/Abstract] OR "happiness"[Title/Abstract] OR "life satisfaction"[Title/Abstract] OR "psychiatry"[Title/Abstract] OR "psychopathology"[Title/Abstract] OR "psychiatric symptom*"[Title/Abstract] OR "mental state*"[Title/Abstract] OR "psychological state*"[Title/Abstract] OR "affective state*"[Title/Abstract] OR "psychological distress"[Title/Abstract]) NOT ("older adult*"[Title/Abstract] OR "elderly"[Title/Abstract] OR "child*"[Title/Abstract] OR "mice"[Title/Abstract] OR "animal*"[Title/Abstract] OR “rats”[Title/Abstract])

*2) Ego Networks*

(“ego network*”[Title/Abstract] OR “personal network*”[Title/Abstract] OR “ego-network*”[Title/Abstract]  OR “egocentric network” [Title/Abstract] OR “individual social network*”[Title/Abstract] OR “personal social network*”[Title/Abstract]) AND (“mental health” [Title/Abstract] OR “wellbeing” [Title/Abstract] OR “well-being” [Title/Abstract] OR “positive affect” [Title/Abstract] OR “negative affect” [Title/Abstract] OR “mood” [Title/Abstract] OR “depression” [Title/Abstract] OR “stress” [Title/Abstract] OR “schizophrenia” [Title/Abstract] OR “anxiety” [Title/Abstract] OR “relapse” [Title/Abstract] OR “disorder” [Title/Abstract] OR “bipolar” [Title/Abstract] OR “happiness” [Title/Abstract] OR “life satisfaction” [Title/Abstract] OR “psychiatry” [Title/Abstract] OR “psychopathology” [Title/Abstract] OR “psychiatric symptom*”[Title/Abstract] OR “mental state*”[Title/Abstract] OR “psychological state*”[Title/Abstract] OR “affective state*”[Title/Abstract] OR “psychological distress” [Title/Abstract] OR “psychology” [Title/Abstract]

 OR “mental distress” [Title/Abstract]) AND (“longitudinal*” [Title/Abstract]

OR “time-series” [Title/Abstract] OR “time series” [Title/Abstract] OR “dynamic*”[Title/Abstract] OR “repeated”[Title/Abstract] OR “change*”[Title/Abstract] OR “long term”[Title/Abstract] OR “long-term” [Title/Abstract]) NOT (“older adult*”[Title/Abstract] OR “elderly”[Title/Abstract] OR “child*”[Title/Abstract] OR “mice”[Title/Abstract] OR “animal*”[Title/Abstract] OR “rats” [Title/Abstract])
